# Incorporating Morphological Evaluations into Breeding Soundness Examinations for Female Dogs

**DOI:** 10.3390/ani15142045

**Published:** 2025-07-11

**Authors:** Dane Wells Schwartz, Jonah Kvernum, Naomie Macias, Muhammed Salman Waqas, Michela Ciccarelli

**Affiliations:** 1 Department of Clinical Sciences, College of Veterinary Medicine, Auburn University, Auburn, AL 36832, USA; dws0048@auburn.edu; 2 Department of Veterinary Clinical Sciences, College of Veterinary Medicine, Washington State University, Pullman, WA 99164, USA; jonah.kvernum@wsu.edu (J.K.); johannah.macias@wsu.edu (N.M.); salman.waqas@wsu.edu (M.S.W.)

**Keywords:** coat color, infertility, bitch, breed, genetics, diabetes mellitus, gestation, pregnancy, French bulldog, mammary gland

## Abstract

This article highlights the importance of evaluating the morphological characteristics of female dogs during breeding soundness examinations (BSEs) to assess their reproductive potential and ensure the health of future offspring. Key traits considered in this evaluation include body type, body condition score, skull size and shape, mammary gland conformation, and coat quality. Each of these factors plays a significant role in the health and fertility of breeding females. For example, deviations from breed standards in body size can lead to complications during pregnancy and whelping, while an inappropriate body condition score may disrupt hormonal balance and reproductive cycles. This review also addresses concerns related to brachycephalic breeds, whose conformation can contribute to respiratory and reproductive issues. Additionally, assessing vertebral and pelvic conformation is crucial to prevent dystocia and other complications during delivery. By systematically evaluating these morphological traits, veterinarians can promote ethical breeding practices that prioritize the welfare and genetic health of both breeding females and their puppies. Therefore, integrating comprehensive morphological evaluations into BSEs is essential for responsible dog breeding management, ultimately supporting better reproductive outcomes and healthier future generations.

## Simple Summary

Dog breeding practices have evolved in the last century, shifting from an emphasis on functional traits to prioritizing aesthetic considerations as dog shows gained popularity. The formal establishment of kennel clubs marked the rise of breed standards prioritizing physical appearance, which inadvertently led to the proliferation of breeds with health issues stemming from genetic defects. Selective breeding and inbreeding have led to significant health problems, necessitating initiatives to enhance genetic diversity and breeding ethics that prioritize health over appearance. While the practice of crossbreeding, leading to designer breeds like Labradoodles, may offer increased genetic diversity and potentially fewer health issues, purebred dogs, bred to conform to specific written breed standards, provide predictability in terms of traits and are well-established regarding their characteristics and standards. This review emphasizes the importance of rigorous veterinary assessments of morphological traits during breeding soundness examinations, as deviations from breed standards or newly introduced characteristics can negatively impact reproductive health and progeny wellbeing. As dog breeding continues to evolve, the integration of ethical principles and health-focused practices becomes essential for safeguarding the future of canine companions.

## 1. Introduction

Since the domestication of dogs, humans have played a significant role in developing and differentiating dog breeds by managing their reproduction and intentionally breeding for specific traits [1]. Initially, the selection process centered on functional characteristics, and dogs were chosen based on their suitability for roles such as hunting, herding, and guarding. As dog shows gained popularity alongside industrialization and urbanization, dogs evolved into status symbols and integral family members for their owners. Concurrently, the emphasis on breeding shifted from practical traits to appearance, with breed standards carefully documenting these traits. Selecting for the most appealing characteristics in a dog influences a collection of genes, leading to differences in several traits simultaneously (pleiotropy) [2]. As a result, altering one gene through selective breeding can lead to unforeseen changes in other characteristics, potentially resulting in severe developmental disorders and health issues.

**Figure 1:**
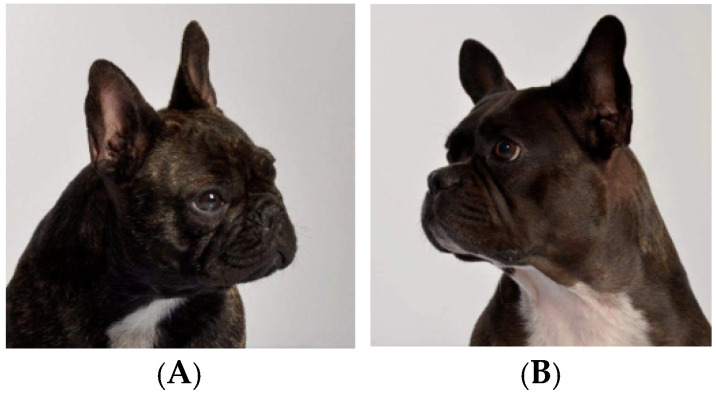
Typical French bulldog, with a short snout (**A**). French bulldog with a longer muzzle from Hawbucks French Bulldogs (**B**).

French bulldogs exemplify the negative consequences that can arise from selective breeding and inbreeding, primarily leading to reduced genetic diversity and significant health issues. Various initiatives have been launched to enhance the breed’s genetic diversity and well-being. For example, a kennel in the Netherlands (https://hawbucks.nl/en/ (accessed on 8 July 2025)) successfully improved the health and longevity of its line of French bulldogs by implementing strict guidelines in its breeding program, including examining for hip and elbow dysplasia, patellar luxation, cardiac disease via ultrasonography, degenerative myelopathies via imaging, and CT scan and MRI abnormalities of the head and spinal cord (https://hawbucks.nl/en/health-tests/#tab-id-1 (accessed on 8 July 2025)) as well as selecting for a French bulldog that is less predisposed to obstructive airway syndrome (Figure 1).

Crossbreeding has been employed to address these health issues and preserve desirable characteristics. The breeds that result from this intentional crossbreeding are known as designer or hybrid breeds. Labradoodles are among the original designer dog breeds. Additional designer breeds include Cockapoos (Cocker Spaniel and Poodle), Schnoodles (Schnauzer and a Poodle), Puggles (Beagle and a Pug), and Shih-poos (Shih Tzu and a Poodle). Designer breeds do not have official breed status. The DNA makeup of Labradoodles and Australian Labradoodles has been studied. Labradoodles are typically a combination of 50% Labrador Retriever and 50% Poodle, while Australian Labradoodles are predominantly Poodle. As anticipated for a designer breed, Labradoodles have a higher genetic diversity than Labrador Retrievers and Poodles [3]. Nevertheless, Australian Labradoodles can suffer from health issues, including orthopedic problems (such as patellar luxation), eye diseases (such as progressive retinal atrophy), and bleeding disorders (such as Von Willebrand disease) [3]. 

The Fédération Cynologique Internationale (FCI) currently recognizes 344 breeds, while the American Kennel Club acknowledges 193 breeds and the Kennel Club in the UK lists 210 breeds. But what characterizes a dog breed? While a dog’s lineage can be identified by its physical characteristics—such as coat color, body shape and size, leg length, and head shape—the notion of a breed has been officially described by both dog enthusiasts and genetic researchers. While it is essential to understand the general standard traits for each breed, the veterinarian should also be able to identify anomalies unrelated to the breed or exacerbated by inbreeding. Veterinarians have the right to refuse assistance in breeding dogs if it compromises animal welfare. While some breeders and breed clubs work with veterinarians to address these issues, the rising popularity of specific breeds and designer mixes has led to an increase in production by irresponsible breeders, self-proclaimed “dog-breeding specialists”, and puppy mills. These breeders frequently overlook the importance of genetic counseling, prioritizing profit over the well-being of the animals by focusing solely on the trends of popular breeds and mixes.

**Figure 2:**
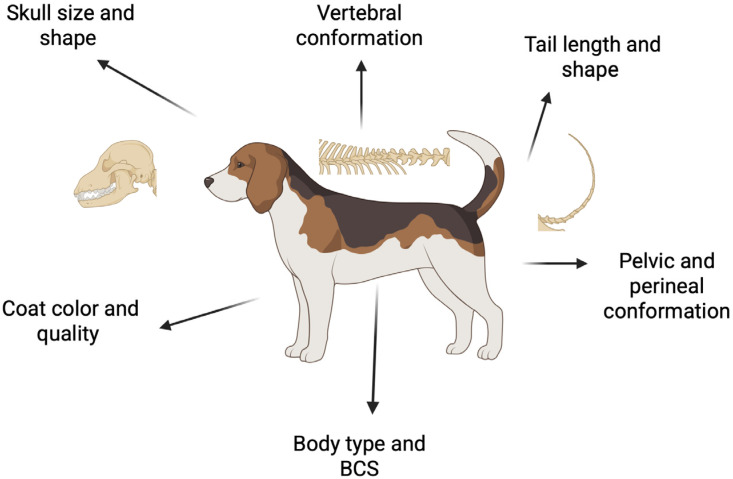
Schematic of morphologic characteristics to include in the routine breeding soundness exam. Created in BioRender. Ciccarelli, M. (2025) https://BioRender.com/7q5tv2d.

When contemplating dog breeding, veterinarians must meticulously assess a variety of morphological traits to ensure not only compliance with breed standards but also the health, fertility, and overall well-being of the progeny. This review outlines the essential morphological characteristics to consider during a breeding soundness examination of a female dog (Figure 2). It emphasizes how deviations from breed standards and the presence of novel traits can compromise the dam’s fertility and the health of future offspring. Additionally, a summary of the components included in a standard breeding soundness exam for a bitch is provided.

## 2. Breeding Soundness Examination

The breeding soundness examination (BSE) is typically performed before breeding. Most are performed on males due to the ease of semen collection and evaluation; however, the female BSE can provide insight into animals with specific fertility problems [4]. It is important to remember that the BSE is a snapshot in time, and fertility and health outcomes can change. A thorough history must be obtained, which includes details about the animal’s signalment, reproductive history, nutrition, vaccination status, and medical background. Understanding the owner’s goals for intended breeding plans is also essential. Genetic counseling plays a crucial role in the BSE to ensure that the animal selected as a potential breeder is free from genetic mutations that could be passed on to future generations. Ideally, breeders should have received Orthopedic Foundation for Animals (OFA) clearances and been tested to ensure they are free from the genetic diseases typical of that breed. As a general guideline, the veterinarian should request the health clearances deemed necessary by the Orthopedic Foundation for Animals (OFA) for the specific breed. The OFA website outlines detailed health screening recommendations for each breed through its CHIC (Canine Health Information Center) program, which serves as an excellent reference for determining appropriate health testing for breeding stock. A thorough physical examination must be performed systematically. The authors prefer to take a rectal temperature last, as this seems to be the most uncomfortable portion of the examination. Each body system is systematically examined. Particular attention should be paid to physical abnormalities that are heritable or may reduce fertility. It is the authors’ opinion to auscultate the thorax first. Cardiovascular anomalies may be first detected during general auscultation, with further diagnostics warranted when found or in at-risk breeds. For example, in Bull-mastiffs and Newfoundland’s, an echocardiogram is indicated before breeding because these breeds are known for a high incidence of subvalvular aortic stenosis [5]. A comprehensive ophthalmic examination by a board-certified veterinary ophthalmologist can be performed through the OFA Companion Animal Eye Registry (CAER). Certification from CAER provides breeders with information on heritable eye conditions, which can inform breeding decisions.

**Figure 3:**
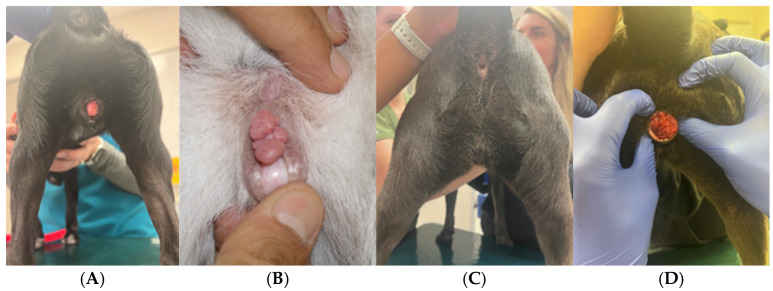
Vaginal masses: hypertrophic clitoris (**A**); transmissible venereal tumor (TVT) (**B**); same dog, without the external appearance of TVT (**C**), and during a vaginal exam with exteriorization of the TVT (**D**).

**Figure 4:**
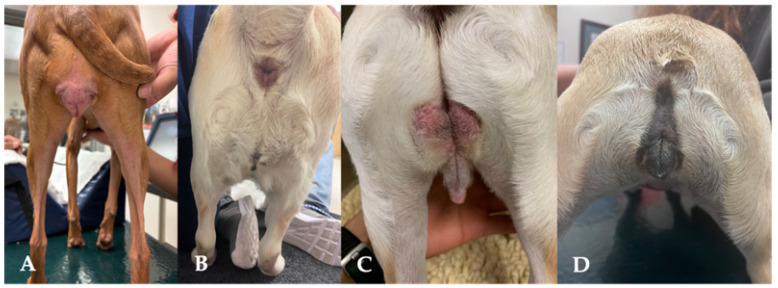
Perineal conformation. Normal perineum of a bitch in heat (**A**). Recessed vulva of a bitch in proestrus (**B**). Abnormal perineal appearance due to DSDs (**C**,**D**).

Particular attention should be paid to mammary gland conformation, including number of teats, masses, and regional lymph nodes in all bitches. Older intact females may be at increased risk for mammary neoplasia [6]. Any enlargement of the mammary glands should be investigated. Additional indicated diagnostic tests include ultrasonography and fine-needle aspiration. Breeding may be postponed if an enlargement is present. Vaginal digital palpation should be performed in maiden and older females to exclude the presence of the hymen and strictures, respectively. Vaginal cytology is performed next to help identify the stage of the estrous cycle. Females in proestrus or estrus are generally more tolerant of this evaluation than those in anestrus [4]. Careful restraint should be applied, especially in maiden females. Abnormalities of the vagina, including stenosis or masses, do not always present with clinical signs (Figure 3) [7]. If abnormalities are found, surgical correction can result in good fertility and successful whelping [7]. Attention should also be placed on the clitoris and clitoral fossa, as disorders of sexual differentiation (DSD) can result in an abnormal perineal conformation (Figure 4) [8]. If found, further diagnostics are recommended, as most animals with DSD are infertile. Transabdominal ultrasonography is also often included in BSEs to exclude fluid accumulation in the uterus and visualize the ovaries. 

A *Brucella canis* (*B. canis*) negative test is required before breeding any dog. *Brucella canis* is a zoonotic, Gram-negative, intracellular bacterium. The devastating consequences of *B. canis* infection include reproductive disruption as well as other systemic diseases. Infected dogs do not always exhibit outward signs of disease, which can lead to the further spread of the infection. Pregnant bitches can experience early embryonic death (during the first 20 days in gestation) and subsequent subfertility or late-term abortion (45–55 days in gestation) [9]. Clinical signs in males are beyond the scope of this paper. Although many diagnostic testing options are available, they have limitations. However, when used together, they can provide a comprehensive diagnosis (https://cdn.ymaws.com/www.theriogenology.org/resource/resmgr/brucella_canis_position_stat.pdf (accessed on 7 July 2025)). 

## 3. Morphologic Characteristics

The evaluation should be systematic from the moment the dog enters the exam room. It should begin with an assessment of general body conformation, body condition score (BCS), coat condition, and color. The evaluation will progress methodically from the head to the tail, covering aspects such as skull size and shape, ocular condition, vertebral conformation, and tail conformation. Finally, characteristics influencing the animal’s reproductive potential should be assessed, including the pelvic area, perineal region, and mammary glands.

### 3.1. Body Type 

The size of a dog is a critical factor that can influence its reproductive health and that of its offspring. Recognized breeds range from toy to giant, with interbreed ranges in size, which can create significant health challenges. For example, breeding two large breed dogs may lead to pregnancy complications and dystocia due to the large litter size when compared to smaller breeds [10]. On the other hand, breeding smaller dogs can sometimes lead to a higher incidence of congenital conditions and may also influence fitness and stamina. When dogs are bred for small stature, they may have a reduced lung field and short extremities, making extraneous activity difficult. Veterinarians need to evaluate the size of breeding candidates and their compatibility to reduce the risk of health problems that could affect the progeny.

Toy breeds are among the smallest dog breeds, typically weighing under 5 kg. Some of these dogs, such as the Chihuahua and the Yorkshire Terrier, have, among other problems, a higher-than-average incidence of congenital hydrocephalus. An even more extreme example is the Cavalier King Charles spaniel. Their brain size has remained that of a bigger dog, and their skull has become smaller and domed. In many Cavalier King Charles Spaniels, it leads to Chiari-like malformation and syringomyelia, complex conditions affecting the brain and spinal cord [11]. In mild cases, the only visible symptom can be the scratching of the back of the neck, while, in severe cases, dogs vocalize in discomfort. The Chihuahua’s large, domed head is prone to malformations. The incomplete development of fibrous membranes between cranial bones, termed persistent fontanelles, is common in the Chihuahua [12]. Persistent fontanelles may be associated with other conditions such as syringomyelia [12]. 

The size of the breed has a significant impact on litter size. Miniature breeds have smaller litter sizes compared to giant breeds (mean: 3.5 vs. 7.1) [13]. In one Swedish study, miniature or giant breeds were at the highest risk for dystocia when compared to other breeds [14]. Of these breeds, the West Highland White Terrier and Bernese Mountain Dog are the most prominent. Additionally, in the Bernese Mountain dog, the risk of C-section decreases with parity but increases with age [15]. Regardless of the breed, 63.8% of bitches with dystocia required a Cesarean (C-section) [14]. 

Artificial selection for high growth rates in large breeds has led to an increased incidence of developmental diseases, which in turn reduce longevity [16]. Giant dogs such as the Great Dane, Newfoundland, St. Bernard, and others have average lifespans significantly shorter than smaller breeds [16,17].

### 3.2. Body Condition Score

Body condition scoring is a subjective assessment of subcutaneous fat, where animals are assigned a numerical score on a scale from 1 to 9. The scores reflect the amount of fat, or condition, that an evaluator can feel when palpating the thorax and topline. A score of 1 indicates that the animal is emaciated, 5 signifies an ideal body condition, and 9 indicates obesity [18]. There is significant interaction between the hypothalamic–pituitary–gonadal axis and body fat. Leptin, a protein hormone produced by fat cells, acts on kisspeptin neurons within the hypothalamus and influences the pulsatility of gonadotropin-releasing hormone (GnRH). The onset of puberty, which marks the beginning of cyclicity in female dogs, is closely related to the animal’s body fat levels. Small-breed dogs reach puberty at an earlier age compared to larger breeds [19]. Dogs with a body condition score (BCS) of less than 5 are considered thin and tend to have a decreased release of leptin. This decrease can delay the onset of puberty and potentially reduce their breeding potential.

Obesity is a prevalent issue in canines [20]. In one extensive study, breeds like the Labrador retriever and herding breeds were found to be at greater risk for developing excess body weight, which was associated with a range of comorbidities, including orthopedic and endocrinologic disorders [20]. Specifically, overweight dogs are at increased risk for conditions such as type 2 diabetes, osteoarthritis, hypertension, and even certain forms of cancer, all of which can further compromise reproductive performance and overall health. The associations between obesity and neoplasia are complex and require further research [21]. Additionally, increased fat deposition in dogs has been linked to other hormonal imbalances, particularly involving thyroid function. Hypothyroidism often results in obesity. Diagnostically, hypothyroidism is indicated by elevated thyroid-stimulating hormone (TSH) levels alongside low or normal free thyroxine (fT4), thyroxine (T4), or triiodothyronine (T3) levels [22]. Such hormonal deficiencies may impair ovulation, weaken luteal function, and lead to early embryonic loss, ultimately causing subfertility or infertility. In hypothyroid female dogs, uterine contractions may be reduced, increasing the risk of difficulties during labor (dystocia) and perinatal mortality [23,24,25]. Although the effects of hypothyroidism on fertility are well-documented in humans [26,27], similar evidence in dogs is still insufficient. Veterinarians must carefully evaluate all potential causes of reproductive failure, including infectious, genetic, and husbandry factors, before diagnosing and administering thyroid hormone treatments.

Autoimmune thyroiditis also needs to be considered, as it is a common cause of primary hypothyroidism in dogs. This immune-mediated destruction of the thyroid gland will lead to a decreased production of thyroid hormones, which can interfere with normal reproductive function. Many breeds predisposed to autoimmune thyroiditis, such as Doberman Pinschers, Golden Retrievers, and Boxers, are specifically recommended or required to undergo thyroid function testing as part of their breed’s OFA (Orthopedic Foundation for Animals) health screening standards. This further underscores the importance of referencing breed-specific health testing guidelines, such as those outlined in the OFA’s CHIC (Canine Health Information Center) program, to ensure that breeding candidates are not only phenotypically sound but also free from inheritable endocrine disorders. 

Other complications linked to high BCS/obesity in domestic animals are the metabolic disorders occurring during pregnancy. The physical space that growing fetuses occupy, especially in the last third of gestation, minimizes space for gastric fill, decreasing appetite and digestible nutrients. Reduced carbohydrate ingestion requires fat mobilization and processing through beta oxidation in the liver. The liver eventually reaches its maximum ability to process the mobilized fatty acids, so the production of ketones occurs. Elevated ketones are a cause for clinical signs and the exacerbation of disease due to depression and suppression of appetite in a ketotic animal. Pregnancy toxemia is not common in dogs, but it has been reported [28,29]. Clinical signs of pregnancy toxemia include persistent anorexia, lethargy, and, if severe, clinical signs associated with liver disease may ensue. Well known in small ruminants, the risk for pregnancy toxemia has been associated with poor quality feed, multiple fetuses, and altered body condition (obese–starvation) [30]. 

Obesity is also considered a risk factor for Progesterone-Related Diabetes Mellitus (PRDM). Similar to humans, body fat triggers a state of chronic inflammation, which subsequently leads to insulin resistance (IR) and type 2 diabetes. In general, female dogs have a two-fold higher incidence of developing diabetes mellitus (DM) than male dogs, with 75% of the dogs diagnosed with DM being females. This type of diabetes is caused by progesterone-controlled growth hormone (GH) overproduction and elevated progesterone levels. This condition is observed in pregnant bitches as well as females during diestrus. Insulin resistance is more severe in pregnant bitches than in those during diestrus, with an increase in clinical signs noted at the end of gestation. GH is known to counter-regulate insulin production and reduce insulin receptor density in cell membranes. Progesterone stimulates GH production by the mammary gland, which operates in an autocrine and paracrine manner to promote ductal tissue proliferation and differentiation. However, some GH can be released into systemic circulation, influencing various changes, including endometrial hyperplasia, insulin resistance, and acromegaly. Breeds such as the Swedish and Norwegian Elkhounds are at an increased risk of developing PRDM, underscoring the role of a dog’s breed and genetic background in predisposing them to DM. Importantly, PRDM is reversible when the progesterone influence is removed, confirming the insulin-resistant nature of this disease [31,32,33]. 

Women with gestational diabetes may experience complications such as dystocia, fetal macrosomia, maternal or newborn mortality, and neonatal hypoglycemia. These issues are implied and reported, albeit with limited evidence, in dogs [31,32,34,35]. 

**Figure 5:**
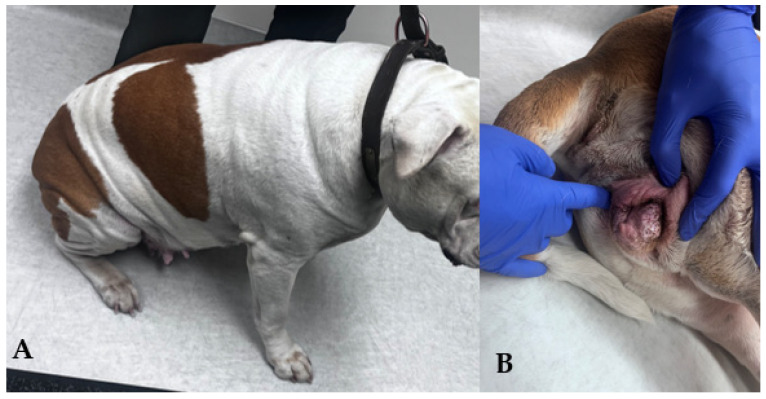
An overweight female American bulldog presented for a breeding soundness exam. Skin fold present in different areas of the body (**A**) and covering her vulva (**B**).

Obesity is thought to lead to the development of additional skin folds in various regions of dogs, including around the perivulvar area. As a result, obesity has been indicated as a potential risk factor for recessed vulvas (Figure 5) [36].

Veterinarians should be able to recognize breeds at risk for DM (Table 1). Preventative measures against the development of DM, including ideal BCS for breeding bitches, should be emphasized to clients. During physical examinations, veterinarians should recognize acromegalic features in intact females, such as skin folds on the ventral neck and over the withers, as well as increased spacing between the teeth. If observed, owners should be discouraged from breeding these animals and instead consider sterilization, as the presence of acromegaly suggests the influence of abnormal quantities of growth hormone. Additionally, the clinician should instruct the owner to look for clinical signs (polydipsia, polyuria, increased appetite with weight loss, lethargy, and potential cataracts) of diabetes mellitus in bitches in diestrus or pregnant. 

### 3.3. Coat Color and Quality

Living alongside humans has diminished the necessity of fur for survival, and the process of domestication has resulted in a greater diversity of coat in terms of quantity, quality, and color. Fur has also been a primary aspect in breeding aesthetic traits, such as the spots on Dalmatians, and practical features, including water resistance in water dogs and the thickness in Arctic breeds. Genetic studies have identified numerous genes associated with coat quantity and coloration, providing valuable insights into health concerns related to specific traits [38,39,40]. For instance, merle-patterned (dark splashes against a lighter pigment) dogs with a homozygous mutation in the PMEL gene have an increased risk of developing health issues, including deafness and blindness, encouraging the prohibition of interbreeding two merle dogs [41]. It is important to note that some dogs can be genetically homozygous for merle (MM) without exhibiting the classic merle coat pattern phenotypically [42]. This hidden merle status underscores the value of genetic testing in breeds where merle is present in the gene pool, as relying solely on appearance can lead to inadvertent high-risk matings. 

Similarly, the lack of pigmentation (hypopigmentation) of the coat and iris is associated with a series of co-occurring nervous–sensory conditions, including sensorineural deafness, iris atrophy, and microphthalmia [43]. 

It is crucial for veterinarians to recognize breeds prone to hearing loss and perform the required testing before breeding. Breeds carrying the merle gene are the Collie, Shetland Sheepdog, Dappled Dachshund, Harlequin Great Dane, Old English Sheepdog, and Norwegian Dunker hound. The piebald gene (white spotting) is present in breeds like the Bull terrier, Samoyed, Greyhound, Great Pyrenees, Sealyham Terrier, Beagle, Bulldog, Dalmatian, and English Setter. Nevertheless, hearing loss has not been documented in all breeds carrying these genes [41]. 

Mutations in three specific genes influence the coat color of Dalmatian dogs. The MITF gene leads to a predominance of white coat, the USH2A gene gives rise to the patchy pattern, and a third gene, currently unknown, is responsible for the evenly distributed, distinct spots. Due to their predominance of white, Dalmatians, like piebald dogs, are at a higher risk of being congenitally deaf. Moreover, they experience a metabolic disorder characterized by elevated uric acid levels (hyperuricosuria), which makes them susceptible to developing urinary stones. This disorder is likely linked to the mutation of the unidentified third gene that dictates the spots in Dalmatians; since all Dalmatians possess spots, they are all affected by hyperuricosuria [44]. In the Dalmatian, altered uric acid metabolism is due to an autosomal recessive gene [45]. Eliminating the disease through breeding is extremely difficult without sacrificing the unique coloration. Nevertheless, specific breeding programs, first initially established by Dr. Robert Schaible through the creation of the Backcross Project, have managed to produce Dalmatians with nearly ideal spotting and a reduced likelihood of urolithiasis by initially backcrossing Dalmatians with Pointers [46]. The Brainstem Auditory Evoked Response (BAER) test is the only accepted method for diagnosing congenital deafness in Dalmatians. The Dalmatian Club of America requests that breeders consider the BAER hearing status of all breeding candidates and their progeny before breeding. Patients must be at least 35 days old before BAER testing can be performed. 

Breeding dogs that deviate from accepted color standards may introduce unpredictability in their offspring, potentially affecting their marketability and health. Therefore, veterinarians must consider both the aesthetic appeal and the genetic implications of coat color before breeding. For instance, a 2021 study examined whether French bulldogs selectively bred for the new “cocoa” phenotype (brown) exhibited any phenotypic effects on thrombocyte function and coagulation capabilities. This is because the gene responsible for the brown coat color in French bulldogs is a variant of another gene found in humans, which is reported to cause oculocutaneous albinism and bleeding disorders due to an absence of platelet granules [47]. This study confirmed that the gene has a significant impact on the function of dense granules in platelets, which could potentially lead to increased bleeding disorders. 

Another concerning trend is the intentional introduction of the merle gene into breeds where the breed standard does not recognize it. This not only increases the risk of merle-associated congenital anomalies, but also often involves incorporating dogs from outside the breed with unknown health backgrounds, solely to introduce a popular or commercially desirable coat color or pattern.

In January 2023, a litter of hairless French bulldogs was reported in the UK. Believed to be a cross between French bulldogs, Pugs, and Chinese Crested dogs, veterinarians are very concerned that these “unique” dogs have an even higher risk of painful acne, dermatitis, sunburn, and skin cancer [48]. 

Another important condition associated with coat type is the dermoid sinus (D.S.) in ridged breeds, such as Rhodesian Ridgebacks, Thai Ridgebacks, and Vietnamese Phu Quoc dogs [49,50]. The dorsal hair ridge with a backward-growing hair phenotype is associated with D.S., a congenital abnormality characterized by the incomplete separation of the ectoderm (skin) and neurectoderm (neural tube) during embryonic development [51,52]. Dermoid sinuses often occur in the dorsal cervical, thoracic, and coccygeal areas. However, in Rhodesian Ridgeback dogs, there is a type that is not connected to the skin, making it challenging to detect through palpation. When the sinus is located near the spine, it can cause neurological issues in the hindlimbs, behavioral changes, and incontinence [53]. This heritable disorder seems to have a complex genetic basis [54], possibly involving multiple gene mutations. Some dogs can be carriers and develop more dermoid sinuses than their littermates, while other lines have few to none. Dogs with D.S. should not be bred. Puppies who have undergone surgery to remove a D.S. are ineligible to compete in conformation shows according to AKC rules. It is crucial to educate owners about these potential issues. Animal welfare cannot be compromised for breeding purposes, as this would perpetuate abnormalities in the canine population. 

The quality of a dog’s coat serves as a reflection of its overall health and well-being. A healthy coat is characterized by shine, density, and cleanliness, all of which are essential indicators of a dog’s nutritional status and health. Breeders should avoid dogs with poor coat quality, as these traits can be hereditary and may indicate underlying health issues, such as allergies, skin infections, or nutritional deficiencies. Furthermore, certain coat types may have specific grooming needs; for instance, long-haired breeds may require more frequent grooming and care, influencing the owner’s ability to maintain proper coat health in the progeny. Dogs with subpar coat quality may also face challenges in reproduction, impacting both the ability to breed successfully and the health of the puppies as coat quality is indicative of nutritional status and overall wellbeing. To the authors’ knowledge, there are no reports of specific reproductive issues associated with coat color as there are in other species [39,55]. 

Notably, designer crossbreeds, such as doodles, often present unique coat challenges due to the unpredictable combination of inherited traits. Their coats can range from curly to wavy to straight, often featuring a soft undercoat combined with a dense or curly outer layer that tends to mat easily. While marketed as low-shedding or hypoallergenic, these coats require frequent brushing and regular professional grooming to prevent painful matting and skin issues. Owners are often surprised to learn that doodles typically have higher grooming needs than either parent breed, making coat maintenance a significant and ongoing commitment, with both financial and physical impacts.

### 3.4. Skull Size and Shape

Brachycephalic dogs are a category of breed characterized by their short nose and wide skull [56]. The desired classic phenotype of a brachycephalic often leads to many issues involving the respiratory, ocular, cardiac, dermatologic, musculoskeletal, thermoregulatory, and reproductive systems [56,57,58,59,60]. Popular breeds within the brachycephalic category include the English bulldog, French bulldog, Pug, Boston terrier, Boxer, and Shih Tzu. In recent years, the popularity of brachycephalic breeds has increased both in the United States and elsewhere, with the French bulldog being the number one most popular breed according to the American Kennel Club in 2024, for the third consecutive year [61,62]. The reproductive efficiency of the breed group is poor, often requiring assisted reproductive techniques (artificial insemination) and delivery by planned C-section. 

Welfare concerns have been raised about the perpetuation of the brachycephalic obstructive airway syndrome (BOAS) through continued breeding, especially with artificial insemination. BOAS includes an elongated soft palate, stenotic nares, everted laryngeal saccules, and everted tonsils [63]. Particularly concerning is the owner’s perception that conditions associated with BOAS are considered normal for the breed [64]. Beyond the scope of this review are the ethical implications of the perpetuation of these animals. Artificial insemination is a common practice in bulldog breeds due to the common physical inability for natural mating. One consideration is the difficulty in breathing and thermoregulation during the copulatory tie [65]. It is not just the short and stout muzzle of brachycephalic dogs that causes respiratory difficulty, as Boxers do not experience the same respiratory difficulty as bulldogs [65]. Respiratory difficulty is often associated with the redundant pharyngeal tissue found in breeds like the bulldog. 

To better evaluate and mitigate these issues in breeding decisions, the Orthopedic Foundation for Animals (OFA) now offers the Respiratory Function Grading Scheme (RFGS), a stress test developed in collaboration with the University of Cambridge. This standardized test includes a brief exercise component followed by the auscultation of breathing patterns to assess clinical signs of airway obstruction. Dogs are assigned a grade from 0 (normal) to 3 (severely affected), with only grades 0 and 1 considered acceptable for breeding. Incorporating this type of functional assessment helps provide objective data on respiratory health and is a valuable tool for breeders and veterinarians seeking to improve the welfare of brachycephalic breeds [66].

In one retrospective study of over 800 dogs, bulldogs represented 17.2% of dogs undergoing C-section [67]. Often, a planned C-section is performed on brachycephalic dogs with managed breeding to reduce neonatal mortality and prevent dystocia. Brachycephalic breeds are at increased risk under general anesthesia due to impaired respiratory function associated with the BOAS abnormalities. The most critical phase of anesthesia in bulldogs is the recovery phase [68]. Proper pre-oxygenation and the selection of reversible anesthetic agents maximize both fetal and maternal outcomes [68].

### 3.5. Ocular Conditions

The initial morphologic evaluation part of the breeding soundness exam should also include ocular abnormalities that could have a genetically heritable component. Bitches showing eye anomalies should be referred to a board-certified ophthalmologist for further evaluations. Below is a list of eye conditions that are inherited and present at birth:

- (CEA) Collie eye anomaly [69].
- (MRD) Multifocal retinal dysplasia [70,71].
- (TRD) Total retinal dysplasia [72].
- (CHC) Congenital hereditary cataract [73].
- (PHPV) Persistent hyperplastic primary vitreous [74].
- (PLA) Pectinate ligament abnormality [75].

Other conditions are inherited but will develop later in life:

- (HC) Hereditary cataract [73].
- (PLL) Primary lens luxation [76].
- (POAG) Primary open-angle glaucoma [75].
- (PRA) Progressive retinal atrophy [77].
- (RPED) Retinal pigment epithelial dystrophy [78].

**Figure 6:**
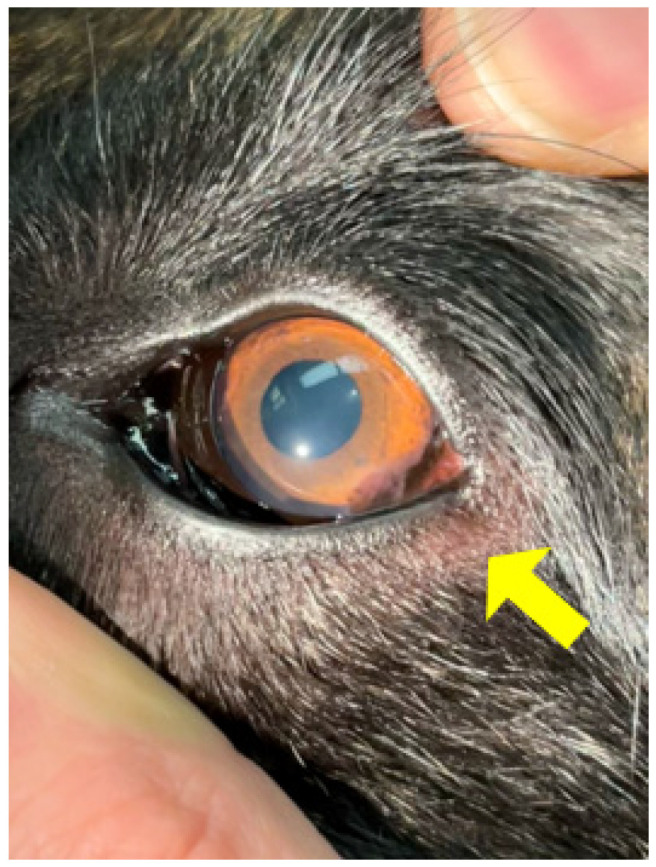
Pannus or chronic superficial keratitis in a German Shepherd (arrow). It was the clinician’s preference to classify the dog as an unsatisfactory breeder due to the potential hereditary component of this eye condition.

There are additional eye disorders for which the hereditary factor remains ambiguous, such as pannus, also referred to as chronic superficial keratitis (CSK) (Figure 6). Although the precise cause is not entirely understood, it is commonly observed in certain dog breeds, especially German Shepherds and Greyhounds. Sunlight exposure and high altitude can worsen the condition.

A genome-wide study in Australian racing greyhounds has identified a region on CFA18 that demonstrates a significant association with the risk of CSK. The haplotype associated with this finding appears to reduce the likelihood of developing the disease. However, this haplotype does not fully account for the disease seen in Australian dogs, indicating that additional genetic loci may also influence the condition in this breed [79]. For comprehensive information on heritable ocular diseases, we recommend “The Blue Book,” which is available online through the ACVO (acvo.org) and OFA (ofa.org/diseases/eye-disease/blue-book/ (accessed 8 July 2025)), as well as the review article by Dr. Kathryn A. Diehl from 2023.

### 3.6. Vertebral Conformation

Congenital vertebral body malformations are commonly diagnosed conditions in brachycephalic, chondrodystrophic breeds. They are usually classified as either defects of segmentation (block vertebra) or defects of formation, which can further be differentiated into aplastic (hemivertebrae) and hypoplastic malformations (wedge-shaped vertebrae, butterfly vertebrae), transitional vertebrae, and neural defects. Most of the time, these issues are incidental findings, yet when a patient presents with myelopathic signs, they are most likely associated with these malformations [80,81].

There is a high prevalence of thoracic congenital vertebral malformations, as described in one study, which is seen in 80.7–87.7% of brachycephalic breeds [81]. Hemivertebrae, defined as a defect in vertebral body formation that results in a deficient body, are seen in 78.0–93.5% of French bulldogs, making it one of the most prevalent disorders [81].

These issues are directly correlated with breeding standards and should be brought to the forefront of efforts to modify and improve the welfare of these animals. In French bulldogs, there is an identified mutation in a gene named DVL2 linked with thoracic and caudal vertebral column malformations [81]. These skeletal abnormalities make copulation and parturition difficult [65]. In a study of orthopedic development in English bulldogs, pelvic torsional deformities were common [82]. These torsional deformities likely impact the ability for natural whelping. 

Breeds like the Basset Hound, Dachshund, and Corgi are characterized by their long bodies and short legs. These breeds are deliberately bred to have a genetic deformity, chondrodysplasia, which contributes to their characteristic appearance. One of the most common diseases of the chondrodysplasia breeds, and in particular the Dachshund, is intervertebral disc disease (IVDD). Type I IVDD is the early degeneration and calcification of intervertebral discs, leading to paraplegia. The radiographic calcification of discs has been associated with the fibroblast growth factor-4 retrogene insertion (FGF4L2). Animals with either the 0 or 1 copy of the FGF4L2 gene have similar and lower radiographic disc calcification scores when compared to those animals with two copies [83]. The calcification of intervertebral discs is associated with an increased risk for disc herniation. Genetic testing for FGF4L2 and radiographic disc calcification scoring (K-number) should be used when deciding on breeding animals, as those animals with two copies are at an increased risk [83]. 

### 3.7. Tail Length and Shape 

Dog tails exhibit a wide variety of lengths and shapes. Some breeds are specifically bred for short stump tails, while others may have tightly curled, corkscrew tails, or may even be born tailless (Table 2). A bobtail appears due to a developmental disorder that affects the vertebrae, resulting from several gene mutations. The most prevalent form of stump tail arises from a heterozygous mutation in the T-box transcription factor *T* gene (TBXT). The breeding of two heterozygotes is predicted to produce 25% puppies with normal length tails, 50% puppies with natural bobtail, and 25% homozygous-affected offspring. Most homozygous offspring are expected to be reabsorbed in utero at an early stage and, therefore, represent a potential 25% reduction in litter size. Unlike in mice and cats, the TBXT gene mutation in dogs is not associated with other growth disorders or health problems [84,85,86]. Some of the breeds that should be tested for this mutation are the Australian Cattle Dog, Australian Shepherd, Brittany Spaniel, Catahoula Leopard Dog, Jack Russell Terrier, Karelian Bear Dog, Pembroke Welsh Corgi, and Pyrenean Shepherd [84,85,86].

**Figure 7:**
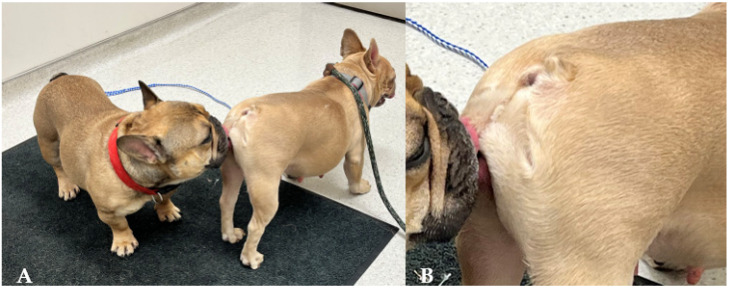
A corkscrew tail in a French bulldog female presented as a teaser (**A**). Corkscrew tail zoomed in (**B**).

Another form of short tail is the so-called “corkscrew” tail, which is found in Boston terriers, French bulldogs, and English bulldogs (Figure 7). This trait is associated with a mutation in the *DVL2* gene, which plays a key role in embryonic development. The mutation can lead to skeletal and cranial abnormalities in affected dogs [87]. In French bulldogs, it has been reported that the selection of the “screw-tailed” phenotype and the degree of this malformation are directly associated with the severity of thoracic hemivertebrae, lumbosacral hemivertebrae, and intervertebral disc degeneration and herniation [80,81]. One study showed that French bulldogs have 9.7 (OR) times higher risk of neurological deficits because of these defects [80]. In English bulldogs, the tail typically consists of several malformed vertebrae, resulting in a compact rosette or spiral shape. At times, the tail may also curve towards the anus, necessitating docking. Boston terriers typically have a very short tail, consisting of only a few vertebrae, which makes them appear nearly tailless. The same gene mutation is also found in other breeds like American Staffordshire Terriers, Staffordshire Bull Terriers, Bordeaux Mastiffs, Old English Bulldogs, and American Bulldogs. These breeds may not consistently exhibit corkscrew tails even with the mutation, suggesting that additional genes are probably at play.

The curly tail is possibly another manifestation of abnormal vertebral development, a condition typical of Pugs. Their tail curls closely along the dog’s back, but the dog is still able to extend it, in contrast to a dog that possesses a corkscrew tail. To date, no genetic mutations have been linked to this type of tail. Selecting sires and dams with more normalized tail anatomy and vertebrae could decrease the prevalence of spinal malformations in this breed as a whole and, therefore, lower the chances of emergencies like disc herniation or protrusion. This is where the breed standard can play a role in evaluating breeding stock in a clinical setting. Understanding the characteristics that deviate from ideal breed standards can help veterinarians identify potentially heritable structural abnormalities during a breeding soundness exam. For instance, the Official standard of the Standard Dachshund states: “*Tail—Set in continuation of the spine, extending without kinks, or pronounced curvature, and not carried too gaily*” [88].

Judges in conformation and veterinarians in clinical practice should be vigilant in palpating for kinked tails. Selective breeding and conscious efforts not to reward dogs who demonstrate extreme phenotypes in breeding shows are relevant ways to improve breed standards [89]. In the show ring, judges will palpate the length of the tail during individual examination, searching for kinks, breaks, or abnormalities that may not be externally visible but indicate structural imperfections. These findings can influence a judge’s decision on whether the dog is a strong representative of the breed and, therefore, suitable for inclusion in the breeding pool. Similarly, in clinical practice, veterinarians should incorporate this detailed evaluation into their breeding soundness exams, not only documenting physical anomalies but also educating owners and breeders about their potential genetic implications. Ultimately, it is the breeder’s decision to breed an animal. It is the veterinarian’s duty to educate on responsible breeding and to provide quality reproductive services that uphold the welfare of the breeding animal. 

### 3.8. Pelvic Conformation and Pelvimetry 

Boston terriers and Scottish terriers revealed abnormalities predisposing to obstructive dystocia when compared with eutocic bitches [90]. Both breeds radiographically revealed a dorsoventrally flattened pelvic canal, predisposing to dystocia caused by fetal-pelvic disproportion [90]. In the normal pelvic inlet, the vertical diameter is typically longer than the horizontal diameter; in both breed groups, either the vertical diameter was longer or they were equal [90]. Additionally, it was found that the pelvic shape in Boston terriers is inherited from both parents, with 26% of the offspring’s pelvic shape explained by the pelvic shape of the mother and father [90]. This highlights the potential benefits of using pelvimetry when assessing breeding females, similar to its application in cattle. By performing radiographs of the pelvis and taking various measurements, it should be possible to estimate whether a female dog is likely to whelp naturally or experience obstructive dystocia. However, since the shape of the pelvis varies among different breeds, more bitches from various breeds need to be measured. These data will help establish an index for each breed to enhance the clinical utility of pelvimetry. Additionally, it could be valuable to compare bitches of the same breed, specifically those who have had normal deliveries with maiden bitches, that have yet to give birth [91].

### 3.9. Perineal Conformation

The evaluation of the perineum should be included in the breeding soundness examination. Standards for the bitch have not been established as they are for the mare [92]. Conditions associated with the conformation of the perineum and vulva can be congenital or acquired, with surgical correction frequently necessary (Figure 4). A recessed or hooded vulva is characterized by a sunken appearance of the vulva, which is often surrounded by excess perivulvar skin. This can make the area look more concealed, potentially impacting both comfort and aesthetics. The surrounding skin may appear to fold inward, creating a distinct contrast between the vulva and its surrounding tissues. Although no causation for the development of a hooded vulva has been identified, it is more commonly seen in spayed dogs compared to intact [36]. Though more commonly found in spayed dogs, a hooded vulva is still seen in breeding stock, and when appreciated, poses a risk for potential reproductive success. Lower urinary tract disease and perivulvar dermatitis are often thought to be increased in dogs with hooded vulvas. However, this has not been shown [36]. Surgical correction by episioplasty (or vulvoplasty) removes the overlying skin fold, giving the vulva more exposure [93]. 

### 3.10. Mammary Gland Conformation

As with other domestic species, the conformational evaluation of the mammary gland should be an essential aspect considered during the selection of breeding dogs. Counting the number of functional glands, assessing nipple shape, palpating the glandular portion, along with ultrasonography, should be included in the breeding soundness exam. 

**Figure 8:**
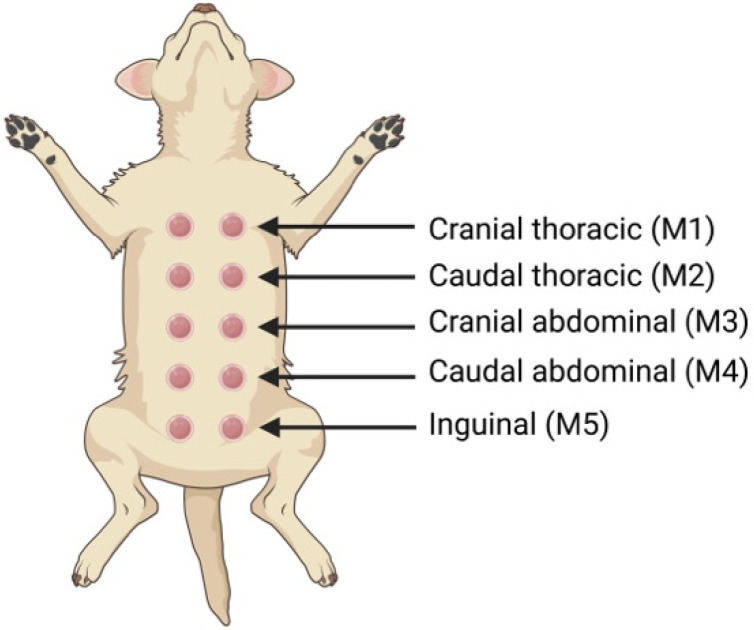
Schematic representation of the anatomical distribution and denomination of the mammary glands in female dogs. Created in BioRender. Ciccarelli, M. (2025) https://BioRender.com/gjg5rzl.

Dogs possess two mammary chains, left and right, with five mammae in each one, with a total of two thoracic pairs (M1 and M2), two abdominal pairs (M3 and M4), and one inguinal pair (M5) (Figure 8). They are not always symmetrical, and four and six pairs of glands have previously been described in some dogs, without any association with the breed [94]. In terms of size, the inguinal pairs are larger than the abdominal pairs, while the thoracic pairs are the smallest [95]. The mammary lymphatic vessels drain into several lymph nodes, which are susceptible to metastasis. The lymphatic drainage of the mammary glands is bilaterally symmetric and variable. The M1 and M2 drain directly into the axillary and sternal lymph nodes. The drainage of the caudal thoracic and abdominal mammary glands can be to either or both the inguinal or axillary lymph nodes. The inguinal gland drains directly into the inguinal lymph node. Communication between left and right sides has also been documented [96]. 

**Figure 9:**
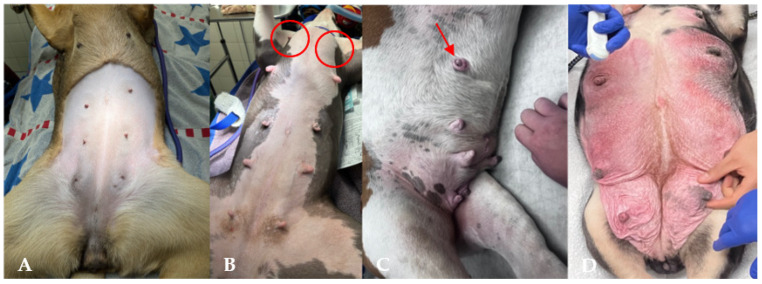
Mammary gland evaluation: Eight mammary glands (**A**). Ten mammary glands with M1 nonfunctional ((**B**), circles). Inverted nipple ((**C**), arrow). Large nipples (**D**).

The number of functional teats is a crucial factor in commercial swine production [97]. As litter size increases, the number of teats must also rise to ensure that all piglets receive adequate nutrition. This principle should also apply to dogs, depending on the expected litter size. Udder and teat conformation is highly heritable in cattle (h2 of udder attachment = 0.2 to 0.3; h2 of teat size = 0.5) [98]; so, enhancing teat and udder quality can be accomplished by avoiding the selection of replacement heifers from dams with poor teat and udder conformation. This should also be considered in dogs, as it can affect the well-being of puppies and future generations. Poor udder and teat conformation can potentially lead to increased puppy morbidity and slower growth since mastitis can develop. Poor teat conformation makes effective nursing challenging, which can result in inadequate milk consumption and insufficient weight gain. Additionally, the inadequate passive transfer of immunity due to insufficient colostrum intake is a predisposing factor for infectious diseases in puppies. In dogs, various teat characteristics can impair the ability to consume enough milk: large, inverted, flat, supernumerary/non-functional, or split or forked teats are all examples of abnormal teat confirmation (Figure 9).

## 4. Conclusions

Despite incorporating morphological assessments into routine breeding soundness examinations (BSEs) and advising owners on requisite health testing before breeding, several challenges persist in optimizing canine reproductive health. A primary obstacle is the heterogeneity in access to comprehensive genetic and health datasets, which limits the capacity to predict and mitigate hereditary conditions accurately. Furthermore, environmental and management variables significantly influence reproductive outcomes, making it challenging to formulate universally applicable breeding guidelines. Future research should prioritize the development of advanced genomic screening technologies and reliable biomarkers capable of precisely evaluating an individual dog’s health status and reproductive potential. Additionally, investigating the effects of environmental enrichment and nutritional modulation on reproductive success may reveal novel strategies for enhancing breed health. Improving our understanding of these multifactorial influences will be essential for designing refined, evidence-based breeding protocols that safeguard the welfare of breeding dogs and their progeny.

**Figure 10:**
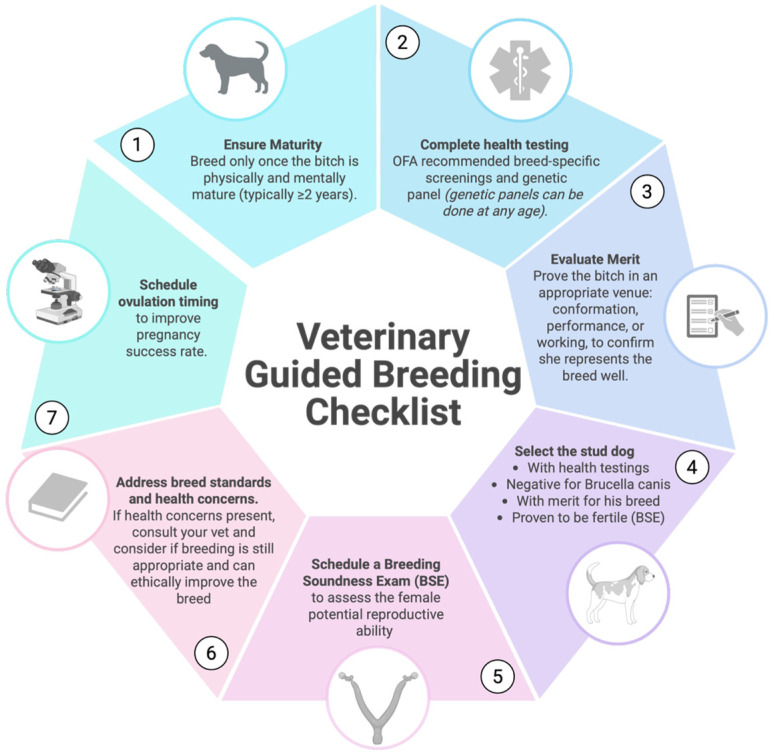
Veterinary-guided checklist for breeders. Created in BioRender. Ciccarelli, M. (2025) https://BioRender.com/bb2jzhx.

By considering the factors discussed in this paper, such as coat color, coat quality, tail characteristics, size, and body condition score, veterinarians can augment their expertise and guide responsible breeding practices. It is incumbent upon veterinary professionals to conduct systematic and informed health and condition evaluations of breeding animals, acknowledging current limitations posed by gaps in scientific data. Abnormalities and potential welfare concerns identified during examination must be communicated effectively to clients. Veterinarians serve an educational role, providing resources and guidance for responsible breeding practices. The client’s receptiveness to this information and their capacity to make informed decisions are crucial. It is not the veterinarian’s role to judge client motivations but to develop personalized breeding programs aligned with the health and welfare of the individual dog and the breed population. To support these efforts, a veterinary-guided breeding checklist has been provided, summarizing essential steps for responsible breeding preparation (Figure 10).

## Figures and Tables

**Table 1:** Dog breeds with a reported predisposition to and protection for diabetes mellitus [37].

Increased Risk for Diabetes Mellitus	Decreased Risk of Diabetes Mellitus
Australian Terrier	Airedale Terrier
Bichon Frise	Basset Hound
Border Collie	Beagle
Border Terrier	Boston Terrier
Cairn Terrier	Boxer
Cavalier King Charles Spaniel	Brittany Spaniel
English Setter	Bulldog
English Springer Spaniel	Cocker Spaniel
Finish Spitz	Collie
Fox Terrier	Dalmatian
Irish Setter	Doberman Pinscher
Keeshond	English Pointer
Miniature and Standard Schnauzer	English Setter
Poodle	German Shepherd Dog
Samoyed	German Short-Hair Pointer
Siberian Husky	Golden Retriever
Swedish Elkhound	Great Dane
Swedish Lapphund	Greyhound
Tibetan Terrier	Irish Setter
West Highlander White Terrier	Norwegian Elkhound
Yorkshire Terrier	Old English Sheepdog

**Table 2:** Categories of tail malformations [80].

Severity of Tail Malformations	Number of Nonmalformed Caudal Vertebra
Nonmalformed	No malformed caudal vertebra
Minimal malformations	>4 nonmalformed caudal vertebra
Moderate malformations	2–3 nonmalformed caudal vertebra
Severe malformations	0–1 nonmalformed caudal vertebra

## Data Availability

No new data were created or analyzed in this study. Data sharing is not applicable to this article.

## Abbreviations

The following abbreviations are used in this manuscript:

BSE	Breeding Soundness Examination
FCI	Fédération Cynologique Internationale
BCS	Body Condition Score
OFA	Orthopedic Foundation for Animals
CHIC	Canine Health Information Center
CAER	Companion Animal Eye Registry
DSD	Disorders of Sexual Differentiation
*B. canis*	*Brucella canis*
C-section	Cesarean section
GnRH	Gonadotropin-releasing hormone
TSH	Thyroid-stimulating hormone
fT4	Free thyroxine
T4	Thyroxine
T3	Triiodothyronine
PRDM	Progesterone-Related Diabetes Mellitus
IR	Insulin Resistance
DM	Diabetes Mellitus
GH	Growth Hormone
BOAS	Brachycephalic Obstructive Airway Syndrome
RFGS	Respiratory Function Grading Scheme
IVDD	Intervertebral Disc Disease
FGF4L2	Fibroblast growth factor-4
BAER	Brainstem Auditory-Evoked Response
DS	Dermoid Sinus
CSK	Chronic Superficial Keratitis
